# Comparing the Drying Characteristics, Phytochemicals, and Antioxidant Characterization of *Panax quinquefolium* L. Treated by Different Processing Techniques

**DOI:** 10.3390/foods14050815

**Published:** 2025-02-27

**Authors:** Meng Li, Shuang Liu, Zhenqiang Wang, Feng Liu, Hongjing Dong, Xuguang Qiao, Xiao Wang

**Affiliations:** 1 Key Laboratory for Applied Technology of Sophisticated Analytical Instruments of Shandong Province, Shandong Analysis and Test Center, Qilu University of Technology (Shandong Academy of Sciences), Jinan 250014, China; lemon982436@163.com (M.L.);; 2 Shandong Academy of Chinese Medicine, Jinan 250014, China; 3 Key Laboratory for Natural Active Pharmaceutical Constituents Research in Universities of Shandong Province, School of Pharmaceutical Sciences, Qilu University of Technology (Shandong Academy of Sciences), Jinan 250014, China; 4 Key Laboratory of Food Processing Technology and Quality Control in Shandong Province, College of Food Science and Engineering, Shandong Agricultural University, No. 61, Daizong Road, Tai’an 271018, China

**Keywords:** *Panax quinquefolium* L., processing, ginsenosides, volatile compounds, antioxidant capacity

## Abstract

American ginseng (AG) has long been used as an ingredient in the food and pharmaceutical industries because of its nutritional and economic value. AG is rich in nutrients, and its quality is greatly affected by how it is processed. However, there is a relative paucity of research on the comprehensive evaluation of different processing techniques of AG. This study evaluated the differences in quality formation and properties of low-temperature softened, blanched, steamed followed by hot air drying, and vacuum freeze-dried AG (LTS-HAD, BL-HAD, ST-HAD, and VFD, respectively). The results demonstrated that AGs treated with VFD had the fastest drying time (85 h) and succeeded in preserving the color and microstructure of fresh ginseng. The contents of ginsenoside Rg1 and Rb1 in LTS-HAD samples were 2.81 ± 0.01 mg/g and 10.68 ± 0.66 mg/g, respectively, which were significantly higher than those in VFD samples (*p* < 0.05). Moreover, ST-HAD samples had an attractive reddish-brown appearance and higher antioxidant activity. Simultaneously, the formation of the ginsenosides Rg6, (S) Rg3, (R) Rg3, Rk1, and Rg5 was discovered. BL-HAD samples had an intermediate quality among the above samples. A total of 58 volatile compounds were identified, including aldehydes (14), alcohols (13), ketones (10), esters (6), terpenes (6), acids (5), and heterocyclic compounds (4). PCA of ginsenosides and volatile components, as well as correlation analysis with color and antioxidant activity, resulted in the identification of different processed products and potential bioactive components.

## 1. Introduction

American ginseng (AG, *Panax quinquefolium* L.), a well-known edible medicinal herb belonging to the Araliacea family, is mainly native to northern North America and southern Canada and is cultivated in Asia [1]. AG contains a variety of active substances, mainly saponins, volatile organic compounds, polysaccharides, and phenolics, which display a variety of health-promoting functions, such as antioxidant, anti-inflammatory, anticancer, and antidiabetic activities, and protects the central nervous system [2,3,4]. In recent years, functional teas and beverages, cosmetics, and health care products based on AG have emerged due to their important nutritional and commercial value. The market demand for AG is increasing, and its cultivation and production are gradually expanding worldwide.

Processing techniques are a critical bond between foods and products and play a key part in achieving the highest possible product quality attributes, such as product appearance, organizational structure, and biological activity [5]. Fresh AG has a high moisture content and is susceptible to microbial contamination and undesirable physiological reactions. In actual production, it must be dried before consumption and storage [6]. However, the long time needed for direct drying is detrimental to the retention of nutritional compounds and flavor components. High-heat blanching and steaming are the traditional primary processing technologies and have been widely used in the processing of Cistanche deserticola and Gastrodia elata Bl. F. glauca S. chow [7,8]. After blanching and steaming pretreatment, the cell wall and cell membrane rupture, the internal water diffusion rate is accelerated. The chemical transformation of effective substances is beneficial for improving the biological activity. In addition, low-temperature softening, which is a common pretreatment processing and storage method in industrial production, is similar to the traditional process of sweating, and changes the texture and hardness of the samples and accelerates the drying rate by redistributing the internal moisture of the samples [9]. In some studies, AGs with steaming treatment have been explored, no reports have been found detailing the characteristics of blanching and low-temperature softening processes.

Processing is a dynamic process of product quality formation, and currently, several analytical methods are used to evaluate the quality of processed AG products. [10]. For instance, Huang et al. [11] evaluated the composition and content changes of ginsenosides in dried AG and red AG using reversed-phase high-performance liquid chromatography coupled with linear ion trap mass spectrometry and screened chemical markers to discriminate between dried and red AG. Guo et al. used ultrahigh-performance liquid chromatography quadrupole-Orbitrap tandem mass spectrometry combined with multivariate statistical analysis to identify a total of 51 saponins in natural drying, steam drying, and vacuum freeze-drying of AG, and five characteristic marker compounds were obtained [12]. In addition, flavor is a critical factor in consumer preference. Cui et al. [13] used gas chromatography–mass spectrometry (GC-MS) and an electronic nose (E-nose) and identified 52 volatile components in American ginseng, mainly including esters, alcohols, acids, aromatics, and aldehydes. Shuai et al. [1] used high-performance liquid chromatography (HPLC) and headspace-gas chromatography-mass spectrometry (HS-GC-MS) to identified 25 volatile compounds and 8 ginsenosides, achieving the discrimination of American ginseng from 4 different regions. However, as typical chemical and analytical methods, the above methods focus on individual types of chemical components rather than synthesizing information on AG. Therefore, there is a need to combine multiple quality attributes and analytical methods, leading to a better understanding of the formation mechanisms of the samples, which can lead to the identification of samples from different processes and the selection of AG samples with optimal activity.

Therefore, the aim of this paper was to evaluate the influences of four processing techniques (low-temperature softened, blanched, steamed followed by hot air drying, and vacuum freeze-dried) on the drying characteristics, physicochemical and microstructural properties, flavor quality, and antioxidant capacity of AG. In addition, the volatile components and ginsenoside components were analyzed qualitatively. Further chemometric and correlation analyses contributed to the identification of different processing processes of AGs. The results of the study contribute to achieving the screening of critical active substances in AG products as well as the quality control of samples subjected to various processes.

## 2. Materials and Methods

### 2.1. Material Preparation

Fresh AG samples were provided by the planting base in Wendeng City (Shandong Province, China). The average diameter, length, and initial moisture content of the samples were 15.00 ± 5.00 mm, 10.00 ± 1.00 cm, and 68.50 ± 2.00% (wet basis, w.b.). After harvesting, the samples were rinsed with high-pressure (90 bar) water and then wiped with filter paper to remove the moisture on the surface of the samples, and the whiskers and lateral roots were removed before processing.

### 2.2. Processing of AG

Based on previous research, fresh intact AG was stored in a specific constant temperature and humidity incubator (BSC-150, Shanghai Boxun Industry & Commerce Co., Ltd., Shanghai, China) at a temperature of 4 °C and relative humidity of 70%. After 5 days of storage, the samples were placed in a laboratory-scale hot-blast air oven (Wujiang Huafei Electric Heating Equipment Co., Ltd., Suzhou, China) with an initial temperature of 30 °C (air speed of 1.0 m/s) and an increase of 2 °C every 24 h. The maximum temperature was 50 °C until the final moisture content was below 13%. LTS-HAD was obtained as the resulting product. Fresh AG samples were placed in hot water (1:5, g/mL) at 80 °C and boiled for 40 min, with continuous rotation. After removing moisture from the surface, the samples were placed in an oven to dry at 50 °C until the final moisture content was below 13%. BL-HAD was obtained as the resulting product. Fresh AG samples were steamed in a normal pressure boiling water steamer for 2 h, put in an oven, and dried at 50 °C until the final moisture content was below 13%. ST-HAD was obtained as the resulting product. Fresh AG samples were pre-frozen at −18 °C for 24 h. The fresh samples were placed into a freeze dryer (Scientz-12n, Scientz Biotechnology Co., Ltd., Ningbo, China) immediately after removal, and the temperature of the cold trap and the vacuum pressure of the drying chamber were set to −40 °C and 60 Pa, respectively. When the final moisture content was below 13%, drying was stopped. VFD was obtained as the resulting product. After drying all samples, the dried roots were vacuum freeze-dried to ensure a consistent moisture content of the samples and stored in a dryer at room temperature pending further analysis.

### 2.3. Drying Curvess

The moisture ratio (*MR*) was calculated using Equation (1) [14]:

(1) $$\mathrm{MR}=\frac{M_{t}-M_{e}}{M_{0}-M_{e}}\;$$

where *M_t_* (g/g, d.b.), *M*_0_ (g/g, d.b.), and *M_e_* (g/g, d.b.) are the moisture content (d.b.) at any time, initially, and at equilibrium, respectively.

### 2.4. Low-Field NMR Relaxation Measurement

Relaxation data were acquired directly from AG with a low field nuclear magnetic resonance (LF-NMR) analyzer (MesoMR23-060H-I, Niumag Corp., Shanghai, China) with a permanent magnet of 0.5 T. The frequency of proton resonance and working temperature were 20 MHz and 35 °C, respectively. The optimized parameters were as follows: pulse sequence = CPMG (Carr–Purcell–Meiboom–Gill), waiting time (TW) = 2000 ms, echo time = 0.2 ms, number of echoes (NECH) = 18,000, and number of slices (NS) = 8.

### 2.5. Determination of Color

The color values of the AG were obtained by an NH310 high-quality portable colorimeter (Shenzhen 3NH Technology Co., Ltd., Shenzhen, China). A D65 light source was used, with a measuring aperture of 4 mm, in the “sample measurement” mode. CIE Lab coordinates are reported as the coefficients L* (lightness), a* (red color), and b* (yellow color). The color difference (∆*E*) was estimated from Equation (2) [15]:

(2) $$∆E={\left[{\left(L^{*}-L_{0}^{*}\right)}^{2}+{\left(a^{*}-a_{0}^{*}\right)}^{2}+{\;\left(b^{*}-b_{0}^{*}\right)}^{2}\right]}^{1/2}$$

where the subscript 0 refers to the color parameters from fresh AG.

### 2.6. Scanning Electron Microscopy (SEM)

The microstructure of the dried AG samples was observed using a SUPRA™55 microscope (Carl Zeiss AG, Aalen, Germany) at a magnification of 200×.

### 2.7. Determination of Volatile Components

AG samples (1 g) were weighed, transferred into a 20 mL headspace vial, and sealed with a magnetic cap. The analyses were completed using an HS-GC-IMS FlavorSpec^®^ Apparatus (Shandong 145 HaiNeng Science Instrument Co., Ltd., Jinan, China). Samples in headspace vials were incubated at 40 °C for 15 min. Then, headspace gas (300 μL) was injected automatically through a heated syringe (50 °C). Extracted VOCs were separated by the gas chromatographic FS-SE-54 column (15 m × 0.32 mm, 0.25 μm; CS-Chromatographie149 Service GmbH, Dürem, Germany) through nitrogen gas (99.99%) at a column temperature of 40 °C. The flow rate started at 2 mL/min for 2 min, increased to 10 mL/min at 10 min, then to 100 mL/min at 20 min, and finally to 150 mL/min at 30 min. The VOCs were ionized in an IMS ionization chamber (300 MBq in positive ion mode), and the ions were driven into a 9.8 cm drift tube with a nitrogen gas flow at 45 °C. Finally, VOC identification using N-ketones C4-C9 as a reference was achieved by comparing the retention index (RI) of the standards and the drift times in the GC-IMS library [16].

### 2.8. Analysis of Ginsenosides

Dried samples of AG were crushed and sieved through a 40-mesh sieve. An accurately weighed 5.0 g of powder was added to 100 mL of 60% methanol, and the samples were ultrasonically extracted for 30 min. The resultant solutions were filtered, concentrated under vacuum, and freeze-dried. Before use, the samples were redissolved in 1 mL of methanol and filtered through a nylon membrane filter (pore size of 0.22 µm). Content analyses were performed using high-performance liquid chromatography-evaporative light scattering detection (HPLC-ELSD) (ACCHROM S6000, Acchrom, Beijing, China). Samples were separated with a Symmetry^®^ C18 Column (5.0 μm, 4.6 mm × 250 mm, Waters Corporation, Milford, MA, USA) at a constant temperature (25 °C) using solvent A (acetonitrile) and solvent B (0.1% formic acid in water) as the mobile phases for gradient elution. The specific elution gradient conditions were as follows: 0–25 min, 19–20% A; 25–60 min, 20–40%. The flow rate was 1 mL/min, and the injection volume was 10 μL. In addition, standard curves were constructed for the corresponding compounds to further calculate the ginsenoside content compared to the results from different samples.

### 2.9. In Vitro Antioxidant Activities

The antioxidant activities of the freeze-dried extract were measured by DPPH radical scavenging, ABTS•+ radical cation scavenging activity, and ferric reducing/antioxidant power (FRAP). DPPH radical scavenging was determined according to Martín-Gómez et al. [17] with a slight modification. Briefly, 100 µL of the sample solutions was mixed with the same volume of DPPH solution (0.08 mM). The solution was shaken and incubated within 30 min at room temperature in the dark, and then, the absorbance was measured at 517 nm (Spark multimode reader, Tecan, Austria). The ABTS•+ radical cation scavenging activity assay was performed according to the previous method reported by Xu et al. [18]. The results were expressed as Trolox equivalents (mmol Trolox/g). The FRAP assay was determined using the method of Luo et al. [19] with FeSO_4_ values as a standard.

### 2.10. Statistical Analysis

All experiments were conducted in triplicate, and the data are expressed as the mean ± standard deviation (SD). One way analysis of variance (ANOVA) was performed with SPSS statistics software (PASW statistics 18, SPSS Inc., Chicago, IL, USA). GC-IMS data were analyzed using GC × IMS Library Search and laboratory analytical viewer (LAV, G.A.S., Dortmund, Germany).

## 3. Results and Discussion

### 3.1. Drying Characteristics Analysis

**Figure 1:**
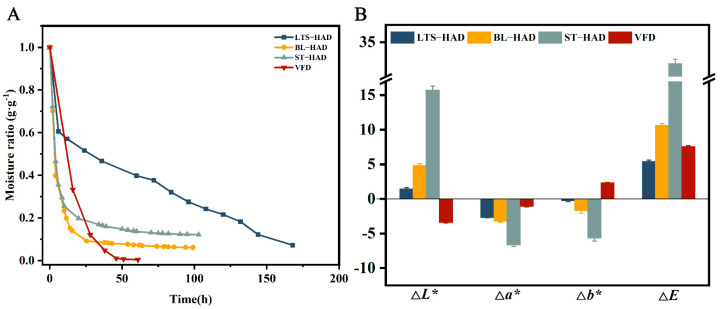
Effects of different postharvest processing methods on the drying curve (**A**), color parameters (**B**).

The drying kinetics of AG procedures were compared with different processes after pretreatment. In Figure 1A, the moisture ratio of each AG progressively diminished as the drying time increased. In the early stages of drying, the moisture content of the samples decreased rapidly; this result indicates that the moisture inside the sample is transferred to the surface more quickly after low-temperature softening, blanching, steaming, and pre-freezing treatments. The drying time varied considerably between the different treatment groups, with VFD samples having the shortest drying time (85 h) and achieving a lower equilibrium moisture content. This is an indication that the vacuum and low temperature conditions in the VFD facilitate the escape and sublimation of moisture within the sample. In addition, BL-HAD and ST-HAD samples have faster drying rates than LTS-HAD samples, which are caused by damage to cell membranes and cell walls by blanching and steaming [20]. Although low-temperature storage accelerates water migration, the lower drying temperature limits the rate of water diffusion.

### 3.2. Water State and Distribution During the Drying Process

**Figure 2:**
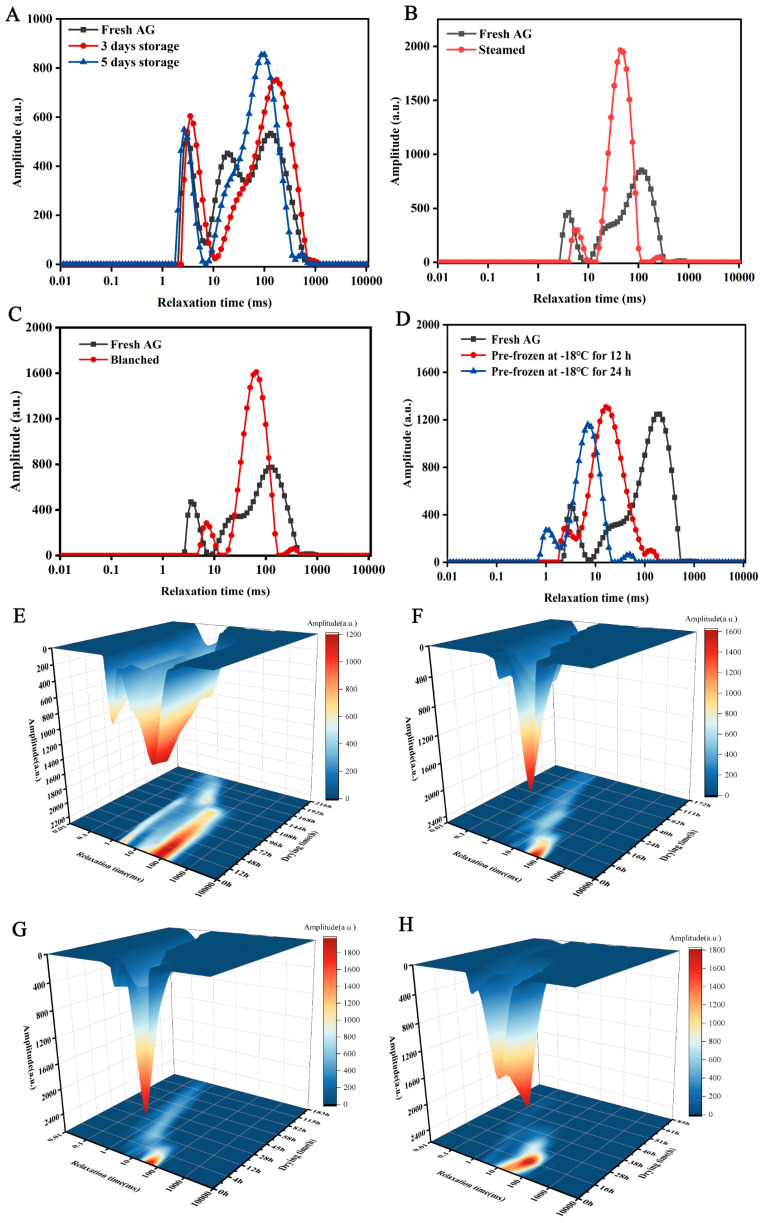
The transverse relaxation time (T2) curves of American ginseng by different pretreatments (**A**–**D**). T2 transverse relaxation time in 3D color map surface image of LTS-HAD (**E**), BL-HAD (**F**), ST-HAD (**G**), and VFD (**H**).

Depending on the inversion atlas (Figure 2A–D), three states of water were observed in the fresh AG samples: free water T23 (100–1000 ms), immobilized water T22 (10–100 ms), and bound water T21 (0.1–10 ms) [21]. The relative content was expressed as peak areas A23, A22, and A21. The water in fresh AG mainly existed as immobilized water and free water, accounting for 43.60% and 40.56%, respectively. In Figure 2A–D, T23 decreased after low-temperature softening, blanching, and pre-freezing but increased after steaming. This indicates that steaming increased the degree of freedom of water, and the presence of water vapor is also possible [22]. A22 was increased in all groups, which suggests that some of the free water was transferred to immobilized water after pretreatment, and in addition, the high temperature led to cell damage and conversion of part of the bound water to immobilized water. As shown in Figure 2E–H, as drying proceeded, the degree of freedom of water gradually decreased, free water was rapidly removed in the early stage, and then immobilized water was more tightly bound to the macromolecules. In addition, only a decrease in A21 was found for all treatment groups of samples after 168 h, 40 h, 28 h, and 46 h of drying, corresponding to LTS-HAD, BL-HAD, ST-HAD, and VFD samples, respectively, which indicates that the bound water was mainly maintained in the late stage of drying.

### 3.3. Color

Color is an important aspect in assessing sensory quality and adding market value for the product. Figure 1B shows the effect of various processes on the color characteristics of AG. Compared to the fresh AG, the LTS-HAD, BL-HAD, and ST-HAD samples had lower L* values (0.20 ± 0.37 ≤ ΔL* ≤ 15.73 ± 0.58), but the VFD samples had a higher value (ΔL* = −3.4 ± 0.08). This suggests that freeze-drying improves the brightness of the samples with its short drying time and lower temperature. Samples from all treatment groups had increased a* values (−6.63 ± 0.24 ≤ Δa* ≤ −1.06 ± 0.09), showing a redder color. This might be due to the blanching and steaming treatments causing the cell structure to be destroyed and intracellular material to leak out, which speeds up the Maillard reaction [23]. Low-temperature storage encourages sugar accumulation, which also accelerates the browning process. The yellowness (b*) of the LTS-HAD, BL-HAD, and ST-HAD samples increased (1.07 ± 0.12 ≤ Δb* ≤ 2.33 ± 0.05), but the VFD was the opposite (−5.67 ± 0.42 ≤ Δb* ≤ −0.23 ± 0.17). Obviously, vacuum freeze-drying technologies exhibit lower enzymatic browning and Maillard response degrees. The total color difference (Δ*E*) represents the distinction between processed and fresh AG. The order of the Δ*E* values for each sample was as follows: ST-HAD > BL-HAD > VFD > LTS-HAD. This result indicates that the high-temperature pretreatment method has a greater effect on the color change of the sample than the low-temperature pretreatment, which is a consequence of the severe disruption of the cellular structure and the acceleration of the browning reaction by the efflux of intracellular material.

### 3.4. Microstructure

**Figure 3:**
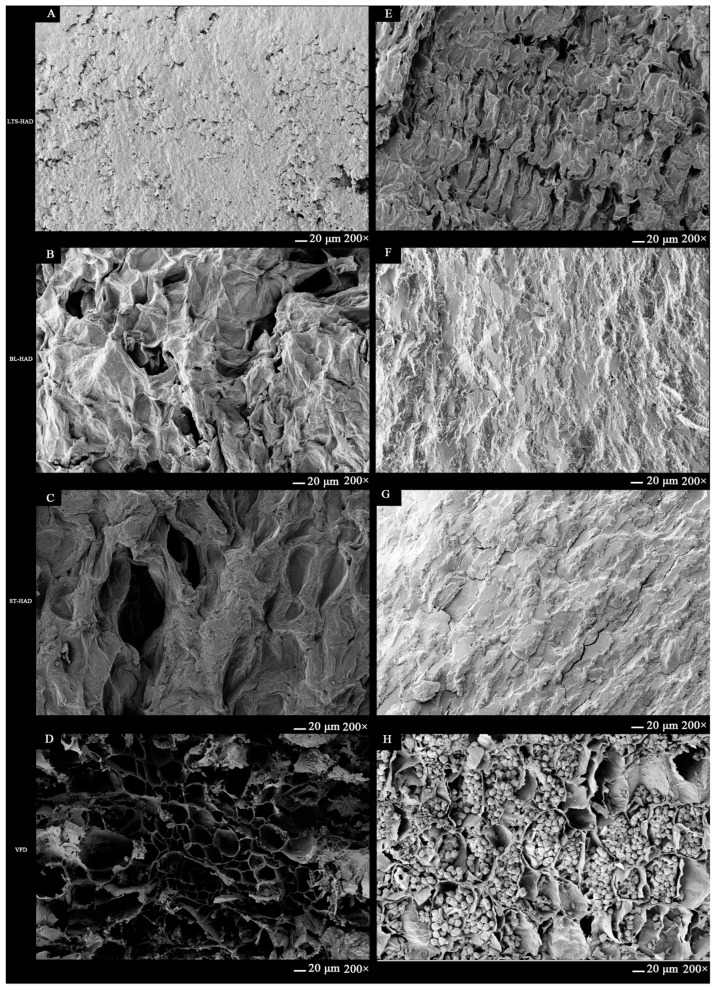
Scanning electron microscope images of American ginseng with different postharvest processing methods. (**A**–**D**) are the samples of LTS-HAD, BL-HAD, ST-HAD, and VFD cross-sections; (**E**–**H**) are the longitudinal sections.

The impact of various treatments on the microstructural modifications of AG was examined using SEM. The VFD samples (Figure 3D,H) had loose honeycomb-like porosity structures, showed a well-preserved cell structure with a distinct vascular bundle, and had many starch granules, which were comparable to those in fresh AG [24]. The LTS-HAD (Figure 3A,E) samples had a coarser structure, with more cracks and distributed starch grains. The BL-HAD (Figure 3B,F) and ST-HAD (Figure 3C,G) samples, however, showed a tight structure, exhibiting distorted histomorphology and collapse due to volume shrinkage; this was caused by the preparation steps of blanching and steaming, which caused cell membranes to rupture and starch to gelatinize, resulting in a gel layer on the surface and increasing the tightness of the samples. As a result, the various processing methods modified the internal morphology of the AG samples, which also impacted their textural characteristics.

### 3.5. Volatile Compounds

**Figure 4:**
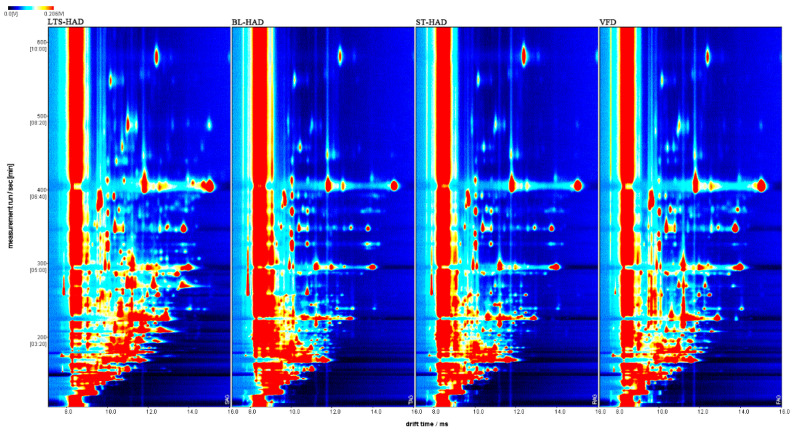
GC-IMS 2D-topographic plots of American ginseng with different processing methods.

Based on the topographic plots by HS-GC-IMS, the various treatments altered the scent characteristics of AG. Each dot on the plots indicates a VOC, with red signals for higher concentrations and white for lower concentrations. As shown in Figure 4, all AG samples were rich in VOCs. However, it was also noticed that the signal of some components faded or disappeared; this showed that the structure and concentration of VOCs in the samples from the various treatment groups differed significantly. The VOCs were characterized using the NIST library by comparing the corresponding IMS drift time and retention index. As shown in Table 1, different treated samples of AG contained a total of 58 fragrance components, including aldehydes (14), alcohols (13), ketones (10), esters (6), terpenes (6), acids (5), and heterocyclic compounds (4). In addition, 38 unknown compounds (U1-38) were also detected. Aldehydes, ketones, and esters were the main VOCs components of AG; this result was consistent with that reported previously.

**Figure 5:**
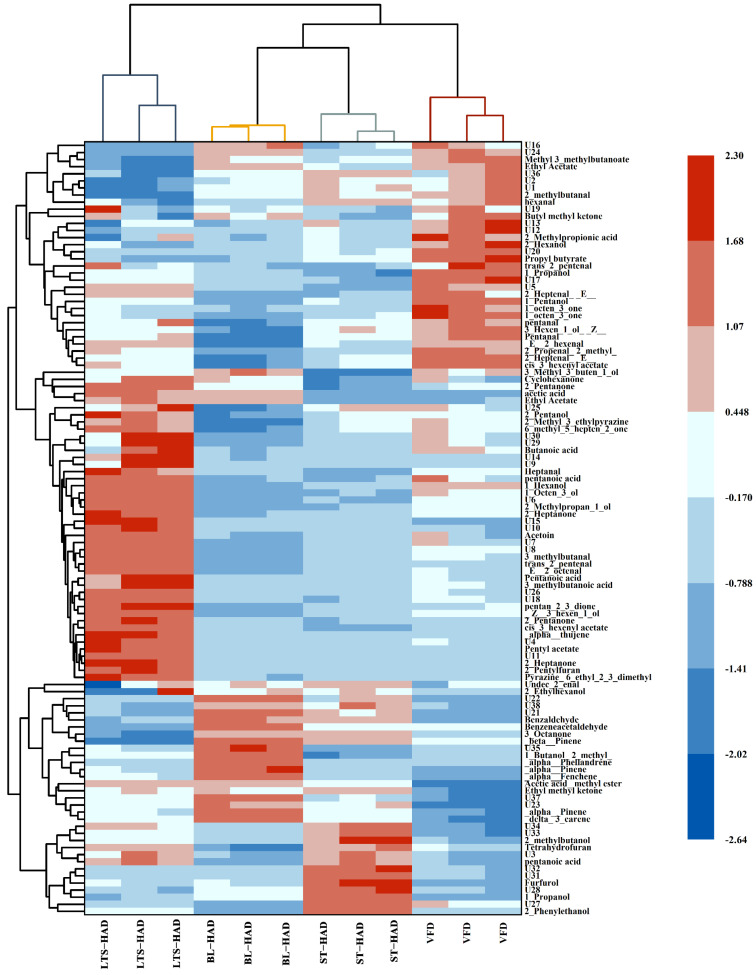
Hierarchical clustering heatmap analysis of volatile compounds in American ginseng with different postharvest processing methods.

A hierarchical clustering heatmap based on Euclidean distance was created to visually confirm the impact of various processing techniques on the volatile components of American ginseng. The results are shown in Figure 5, and the color difference exhibits the VOCs in different samples. Several VOCs, such as 1-octen-3-one, 2-heptanone, and α-pinene, produced multiple signals, which were due to changes in proton affinity that caused the dimers to appear at different drift times. For the samples obtained by the low-temperature softening pretreatment, the LTS-HAD samples contained higher concentrations of aldehydes, ketones, alcohols, acids, and heterocyclic components, such as pentan-2,3-dione, 2-pentylfuran, 2-heptanone, (E)-2-heptenal, 2-pentanone, 1-octen-3-ol, 2-methyl-3-ethylpyrazine, and α-thujene, so the samples mainly had herbaceous, nutty, and fruity aromas. For samples obtained by the cryogenic vacuum process, VFD samples had a higher concentration of alcohols, aldehydes, and esters, with high levels of 1-octen-3-one, propyl butyrate, trans-2-pentenal, and (E)-2-hexenal, which were identified as the main contributors to fresh earthy, green leafy, and fruity aromas [13]. For samples that were pretreated at high temperatures, the BL-HAD and ST-HAD samples contained the highest concentrations of alcohols, aldehydes, and terpenes, and the individual components, pyrazine,6-ethyl-2,3-dimethyl and benzaldehyde, provided distinctive aromas of cocoa and bitter almonds, which were associated with the high-temperature treatment and Maillard reaction. However, BL-HAD samples had a higher concentration of terpenes, such as δ-3-carene, α-pinene, and β-pinene, and may display a sweeter flavor [25]. The ST-HAD samples had higher concentrations of 1-propanol, methyl 3-methylbutanoate, ethyl acetate, and 2-methylbutanal, providing more intense fruit and wine aromas.

### 3.6. Determination of Ginsenosides by HPLC-ELSD

**Figure 6:**
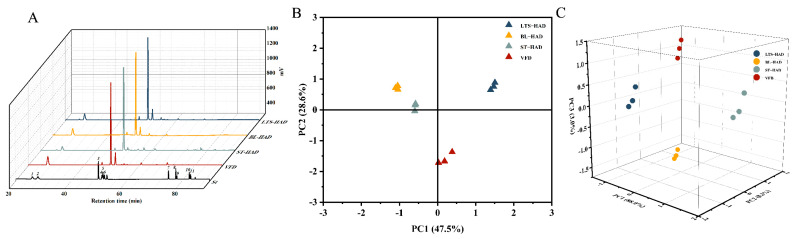
Effects of different processing methods on the HPLC-ELSD chromatograms (**A**). The peak number of chromatograms: 1, Rg1; 2, Re; 3, Rb1; 4, 20(S)-Rg2; 5, Rc; 6, 20(R)-Rg2; 7, Rg6; 8, 20(S)-Rg3; 9, 20(R)-Rg3; 10, Rk1; 11, Rg5. Principal component analysis (PCA) for volatile (**B**) and ginsenoside (**C**) components of four processed American ginseng samples.

Ginsenosides are the major pharmacologically active components of AG. The composition and content of eleven major ginsenosides of different process samples were assayed, and the results are shown in Figure 6A and Table 2. All eleven ginsenoside standards were clearly resolved in the chromatogram of Figure 6A, indicating the viability of this method for determining the ginsenoside concentration in AG samples. Ginsenosides Rg1, Re, Rb1, and Rc were detected in all four samples. Compared with the heat treatment groups, the VFD samples contained the highest Re and the lowest Rb1 content at 45.63 ± 2.32 mg/g and 7.05 ± 0.21 mg/g, respectively. In fact, this can be seen as the best retention of the fresh samples. The LTS-HAD samples dried in a gradient had Rg1 and Rb1 contents that were noticeably higher than those of the VFD samples (*p* < 0.05), whereas the concentrations of Re and Rc were lower. This is consistent with the result of Koh et al. [26] that malonyl ginsenosides were demalonylated to form the corresponding ginsenosides during processing. BL-HAD samples can be thought of as a mid-range product between LTS-HAD and ST-HAD samples. After blanching, the main components were still Rg1, Re, Rb1, and Rc, with Rg1 having the smallest content of 0.69 ± 0.03 mg/g. For the ST-HAD samples, Rb1 is present at high concentrations, possibly due to the dramatic loss of malonyl-Rb1 after steaming. In parallel, the large reduction in Rc was the result of its deglycosylation and dehydration to produce Rg3, which was further dehydrated to produce Rk1 and Rg5 [27]. Therefore, as previously reported, the presence of ginsenosides (S)Rg2, (R)Rg2, Rg6, (S)Rg3, (R)Rg3, Rk1, and Rg5 was also found in the ST-HAD samples. The above results showed that the composition and transformation of the primary component ginsenosides are influenced by the processing methodology and conditions.

### 3.7. Principal Component Analysis (PCA)

The samples were divided into three categories according to the results of the hierarchical clustering analysis (HCA), with LTS-HAD belonging to one cluster, VFD to another, and BL-HAD and ST-HAD to a third. To further determine the classification trends of the samples, the VOCs obtained were subjected to PCA, and the results are shown in Figure 6B. PC1 and PC2 accounted for 73.1% of the total variance, demonstrating that the AG sample sets of the 4 processes could be separated. The BL-HAD and ST-HAD samples are closer in relative position, which reflects the similarity among samples. The above results were in accordance with those of HCA. Therefore, it can be speculated that the volatile substances found in AG are closely related to the processing technique. The contribution of individual fractions to the identification of products from different processes was limited, so we also performed PCA for the ginsenoside components. The score is displayed in Figure 6C, and the principal components for PC-1, PC-2, and PC-3 accounted for 88.0%, 8.3%, and 3.0% of the total variables, respectively. As seen from 3D PCA, the four AG samples had clustered into four distinct groups that occupied relatively independent spaces on the distribution map. Four samples of AG samples had significant differences in ginsenoside content, which is consistent with the results presented in Table 2. Therefore, PCA combined with VOCs and ginsenosides provided an efficient discrimination method for AG samples.

### 3.8. Antioxidant Activities

**Figure 7:**
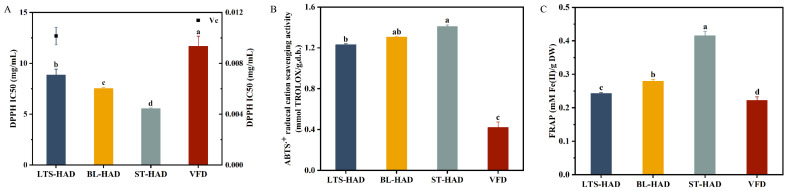
Antioxidant activity of American ginseng subjected to different postharvest processing methods. DPPH radical scavenging activity (**A**); ABTS•+ radical cation scavenging activity (**B**), and ferric reducing/antioxidant power (FRAP) (**C**). Different letters indicate a significant difference (*p* < 0.05).

The antioxidant capacity of the four AG procedures was evaluated by the DPPH ABTS and FRAP methods. As shown in Figure 7A–C, the three methods produced similar results in that marked differences (*p* < 0.05) in the antioxidant activities existed among various AG samples. The ST-HAD samples exhibited the highest antioxidant activity, with DPPH IC_50_, ABTS, and FRAP values of 5.55 ± 0.08 mg/mL, 1.41 ± 0.01 mmol TROLOX/g, and 0.41 ± 0.01 mM Fe(II)/g, respectively, followed by BL-HAD samples. The results were in accordance with the report of Kang et al. [28]. Heat processing releases bound phenolic acids coupled to glucosides or amine functionalities, yielding more conjugated and free phenolic acids. Moreover, the Maillard reaction products after steaming or blanching are another major reason for the enhanced antioxidant capacity of the ST-HAD and BL-HAD samples. Kang et al. also reported that maltol had significant free radical scavenging activity as a byproduct of the Maillard reaction produced by processing ginseng at a higher temperature [29]. Additionally, the LTS-HAD samples showed stronger antioxidant activity than the VFD samples. The possible reason was the changes in the ginsenoside component with a higher antioxidant activity brought on by different processing methods.

### 3.9. Correlation Analysis of Quality Indicators

**Figure 8:**
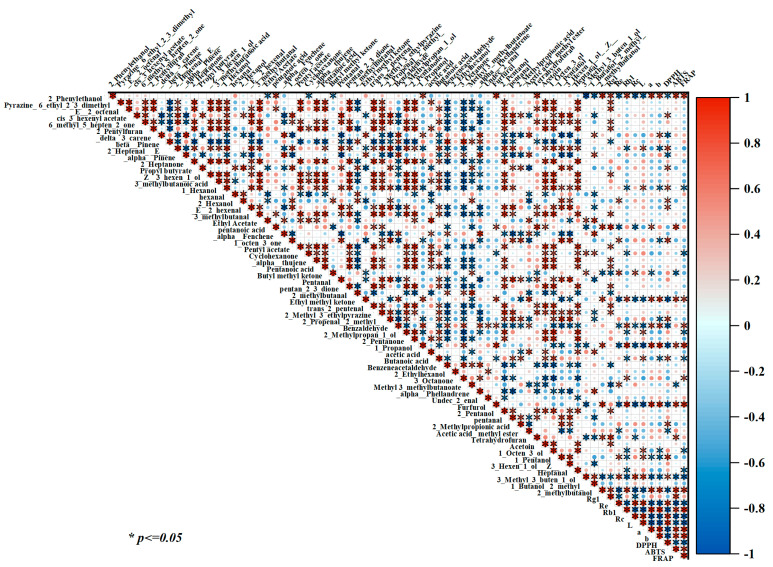
Correlation analysis of volatile compounds, ginsenosides, color, and antioxidant activities.

The relationships among VOCs, ginsenoside content, color parameters, and antioxidant activity were investigated in AG. The strength of the linear relationship between each pair of variables was obtained using the Pearson correlation test. According to the correlation heatmap in Figure 8, volatile components that have a similar odor show a good positive correlation. For example, 2-pentanone, 2-heptanone, and 1-hexanol have a fruity aroma, and their correlation coefficients were 0.93, 0.86, and 0.84, respectively (*p* < 0.05). A significant correlation was found between some VOCs and the color parameters. For example, (E)-2-heptenal, 1-octen-3-ol, 1-hexanol, and butanoic acid were discovered in higher amounts in LTS-HAD samples. These compounds had positive correlations with L* values and negative correlations with a* and b* values (*p* < 0.05). However, compounds, including 2-phenylethanol, benzaldehyde, 1-propanol, furfurol, and 2-methylbutanol, had larger amounts in BL-HAD or ST-HAD samples and exhibited the opposite correlation relationship as the above results. In addition, ginsenosides Re and Rc showed powerful positive relationships with L* values and a significant negative correlation with a* and b* values, but ginsenoside Rb1 demonstrated the opposite trend. The results are consistent with Figure 1B and Table 2, indicating a closely related correlation between the color changes and compositional changes produced by the different treatments. Furthermore, these components can be used as possible markers that efficiently differentiate between different AG processing types. Ginsenosides Rg1 and Rb1 showed significant positive correlations with ABTS radical cation scavenging activity and FRAP with values of 0.72, 0.27, 0.80, and 0.98, respectively. It was negatively correlated with DPPH-IC_50_ at rates of −0.14 and −0.92. However, ginsenosides Re and Rc displayed the opposite trend. In summary, this result indicate that the quality attribute indicators are strongly correlated with one another for AGs processed by different processing methods.

## 4. Conclusions

In this study, a comprehensive analysis of the differences in drying behavior, physical properties, phytochemicals, and antioxidant activity of four processing products of AG was established. The results demonstrated that pretreatment accelerated the rate of diffusion of water within the sample. During the drying process, free water was most affected by the different processes and was the first to be removed; immobilized water dominated the main drying process. Among all treatments, VFD samples better retained the color and microstructure than other processes. ST-HAD samples turned reddish brown and had a tight structure. Deglycosylation, dehydration, and the Maillard reaction produced rich rare ginsenosides and exhibited the strongest antioxidant activity. A total of 58 VOCs were identified in AG, and strategies combining multivariate analysis and correlation analysis were established to identify different processing methods for AG and select processes associated with high antioxidant activity. Overall, although VFD seemed more appropriate to maintain the original quality of AG, ST-HAD showed greater potential for consumption because of the distinct appearance, flavor, and nutritional value. These results provide valuable references for the quality control of processed AG products and the screening of key potential bioactive components.

## Figures and Tables

**Table 1:** Volatile chemical components identified in American ginseng by HS-GC–MS.

NO	Volatiles	Compounds	CAS	Molecule Formula	MW ^1^	ΔRI ^2^	Rt ^3^	Dt ^4^
1	Alcohols	2-Phenylethanol	60-12-8	C_8_H_10_O	122.2	1107	579.341	1.5036
2		1-Octen-3-ol	3391-86-4	C_8_H_16_O	128.2	982.4	376.061	1.1556
3		(Z)-3-hexen-1-ol	928-96-1	C_6_H_12_O	100.2	849.9	258.571	1.5109
4		1-Hexanol	111-27-3	C_6_H_14_O	102.2	871.4	272.67	1.3297
5		2-Hexanol	626-93-7	C_6_H_14_O	102.2	805.4	231.716	1.2882
6		1-Pentanol	71-41-0	C_5_H_12_O	88.1	768.3	212.246	1.2559
7		2-Methylpropan-1-ol	78-83-1	C_4_H_10_O	74.1	626.1	160.094	1.1745
8		3-Methyl-3-buten-1-ol	763-32-6	C_5_H_10_O	86.1	725.5	193.026	1.2497
9		1-Propanol	71-23-8	C_3_H_8_O	60.1	568.4	145.398	1.2504
10		1-Butanol, 2-methyl-	137-32-6	C_5_H_12_O	88.1	738.3	198.692	1.2363
11		2-Ethylhexanol	104-76-7	C_8_H_18_O	130.2	1034.8	447.559	1.4238
12		2-Pentanol	6032-29-7	C_5_H_12_O	88.1	709.6	186.259	1.4624
13		2-methylbutanol	137-32-6	C_5_H_12_O	88.1	748.8	203.455	1.2307
14	Aldehydes	(E)-2-octenal	2548-87-0	C_8_H_14_O	126.2	1058.9	488.852	1.3336
15		(E)-2-Heptenal	18829-55-5	C_7_H_12_O	112.2	955.1	346.521	1.6631
16		Hexanal	66-25-1	C_6_H_12_O	100.2	797.1	227.016	1.5576
17		(E)-2-hexenal	6728-26-3	C_6_H_10_O	98.1	848.8	257.9	1.1795
18		3-methylbutanal	590-86-3	C_5_H_10_O	86.1	649.7	166.593	1.1967
19		trans-2-pentenal	1576870	C_5_H_8_O	84.1	749.7	203.596	1.1034
20		Pentanal	110-62-3	C_5_H_10_O	86.1	697.1	180.982	1.4195
21		2-methylbutanal	96-17-3	C_5_H_10_O	86.1	652.3	167.308	1.4028
22		2-Propenal, 2-methyl-	78-85-3	C_4_H_6_O	70.1	571.9	146.27	1.2179
23		Benzaldehyde	100-52-7	C_7_H_6_O	106.1	959	350.367	1.1488
24		Benzeneacetaldehyde	122-78-1	C_8_H_8_O	120.2	1042.1	459.272	1.2623
25		Undec-2-enal	2463-77-6	C_11_H_20_O	168.3	1351.6	1366.515	1.4929
26		Furfurol	98-01-1	C_5_H_4_O_2_	96.1	825.9	244.341	1.3264
27		Heptanal	111-71-7	C_7_H_14_O	114.2	891.7	287.642	1.323
28	Esters	cis-3-hexenyl acetate	3681-71-8	C_8_H_14_O_2_	142.2	1005	404.259	1.8195
29		Propyl butyrate	105-66-8	C_7_H_14_O_2_	130.2	900.4	294.154	1.6896
30		Ethyl Acetate	141-78-6	C_4_H_8_O_2_	88.1	615.1	157.361	1.3301
31		Pentyl acetate	628-63-7	C_7_H_14_O_2_	130.2	914.5	306.892	1.3119
32		Methyl 3-methylbutanoate	556-24-1	C_6_H_12_O_2_	116.2	766.4	211.695	1.198
33		Acetic acid, methyl ester	79-20-9	C_3_H_6_O_2_	74.1	538.9	139.193	1.1879
34	Ketones	6-methyl-5-hepten-2-one	110-93-0	C_8_H_14_O	126.2	989.5	384.118	1.1741
35		2-Heptanone	110-43-0	C_7_H_14_O	114.2	890.9	286.097	1.2649
36		Acetoin	513-86-0	C_4_H_8_O_2_	88.1	712.7	187.405	1.3258
37		1-octen-3-one	4312-99-6	C_8_H_14_O	126.2	978.8	372.01	1.2796
38		Cyclohexanone	108-94-1	C_6_H_10_O	98.1	899.8	293.627	1.4538
39		Butyl methyl ketone	591-78-6	C_6_H_12_O	100.2	783.5	219.574	1.1917
40		pentan-2,3-dione	600-14-6	C_5_H_8_O_2_	100.1	704.5	184.02	1.2298
41		Ethyl methyl ketone	78-93-3	C_4_H_8_O	72.1	593.2	151.81	1.2416
42		2-Pentanone	107879	C_5_H_10_O	86.1	686.7	177.091	1.3724
43		3-Octanone	106-68-3	C_8_H_16_O	128.2	979.5	372.034	1.3049
44	Heterocycles	Pyrazine,6-ethyl-2,3-dimethyl	15707-34-3	C_8_H_12_N_2_	136.2	1091.5	548.534	1.2306
45		2-Pentylfuran	3777-69-3	C_9_H_14_O	138.2	995.2	390.832	1.2495
46		2-Methyl-3-ethylpyrazine	15707-23-0	C_7_H_10_N_2_	122.2	999	394.564	1.163
47		Tetrahydrofuran	109-99-9	C_4_H_8_O	72.1	626.7	160.059	1.2209
48	Terpenes	δ-3-carene	13466-78-9	C_10_H_16_	136.2	1011.5	413.658	1.2119
49		β-Pinene	127-91-3	C_10_H_16_	136.2	976.4	369.348	1.2124
50		α-Pinene	80-56-8	C_10_H_16_	136.2	935.1	326.38	1.2137
51		α-Fenchene	471-84-1	C_10_H_16_	136.2	949.4	340.657	1.2107
52		α-thujene	2867-05-2	C_10_H_16_	136.2	925.2	316.812	1.6753
53		α-Phellandrene	99-83-2	C_10_H_16_	136.2	997.7	392.725	1.2151
54	Acids	3-methylbutanoic acid	503-74-2	C_5_H_10_O_2_	102.1	869.4	271.327	1.4863
55		pentanoic acid	109-52-4	C_5_H_10_O_2_	102.1	901.2	294.833	1.2202
56		acetic acid	64-19-7	C_2_H_4_O_2_	60.1	636	162.75	1.1493
57		Butanoic acid	107-92-6	C_4_H_8_O_2_	88.1	793.4	225.44	1.3872
58		2-Methylpropionic acid	79-31-2	C_4_H_8_O_2_	88.1	786.7	221.715	1.1665

^1^ Molecular mass. ^2^ Represents the retention index calculated using n-ketones C4–C9 as external references. ^3^ Represents the retention time in the capillary GC column. ^4^ Represents the drift time in the drift tube.

**Table 2:** Effects of different processing methods on the composition of ginsenosides in American ginseng.

Compounds (mg/g)	LTS-HAD	BL-HAD	ST-HAD	VFD
Rg1	2.81 ± 0.01 b	0.59 ± 0.01 e	2.54 ± 0.05 c	0.77 ± 0.01 d
Re	30.97 ± 3.92 b	34.61 ± 1.89 b	20.65 ± 0.50 c	45.63 ± 2.32 a
Rb1	10.68 ± 0.66 c	13.76 ± 0.58 b	23.39 ± 0.76 a	7.05 ± 0.21 d
Rc	12.59 ± 0.91 c	14.62 ± 0.04 b	2.97 ± 0.20 d	20.42 ± 0.27 a
Rg6	nd	nd	0.27 ± 0.03	nd
(S)Rg3	nd	nd	1.84 ± 0.06	nd
(R)Rg3	nd	nd	3.14 ± 0.03	nd
Rk1	nd	nd	0.96 ± 0.14	nd
Rg5	nd	nd	1.53 ± 0.20	nd

Each value is the mean ± the standard deviation (n = 3). Different letters in the same column indicate a significant difference (*p* < 0.05).

## Data Availability

The original contributions presented in this study are included in the article. Further inquiries can be directed to the corresponding authors.
